# Determining the Migration Behavior of Retinal Progenitor Cells in the Embryonic Eye Field of *Xenopus laevis*

**DOI:** 10.64898/2026.05.03.722080

**Published:** 2026-05-10

**Authors:** Randolph L. Grell

**Affiliations:** School of Life Sciences, University of Nevada, Las Vegas, Las Vegas, NV 89154, USA

**Keywords:** EosFP, retinal progenitor cells, eye field, Xenopus laevis, lineage tracing, photoconversion, regionalized differentiation, fate map

## Abstract

Given the critical role of progenitor cells staying within the eye field transcription factor (EFTF) signaling niche for normal eye development, we hypothesized that retinal progenitor cells (RPCs) differentiate within their initial region of inception during eye development. To investigate this, we utilized EosFP, a photoconvertible protein, as a lineage tracer in the model organism *Xenopus laevis*. By employing confocal laser microscopy for photoconversion, we labeled cells within elongated rectangular regions that encompassed both the eye field and the adjacent tissues. In a separate set of embryos, we identified which portions of these rectangular regions harbored cells destined to become part of the mature eye versus those that would form the surrounding tissues, tracing their development from stage 15 to stage 35. This allowed us to create a fate map of the stage 15 embryo using EosFP to accurately locate and label the eye field to address our hypothesis. With the eye field delineated using our lineage tracer, we further employed EosFP to label RPCs within individual quadrants of the developing eye. Tracking these RPCs from stage 15 to stage 35, we observed the retinal cells organizing into three principal layers of cell bodies, mirroring the layered neuroanatomy characteristic of the mature retina. We observed the red-labeled RPCs proliferated but remained predominantly within their quadrant of inception, with no dispersion into other, unlabeled quadrants of the eye by stage 35. These findings corroborate our hypothesis that RPCs undergo differentiation within their initial locations in the eye field. Our study illuminates the cellular dynamics of eye development in *Xenopus laevis* and introduces a novel method for lineage tracing of stem cell populations during embryonic development.

## Introduction

The cells associated with eye development have been traced back to as early as the 16-cell stage in embryonic development in *Xenopus laevis*. Using cell lineage tracing, retinal cell fates can be traced to the dorsal D1.1 and D1.2 blastomeres within the developing embryo (Moody, 1987a). These two blastomeres comprise approximately 48% and 45% of the total retinal cell count seen in the mature retina, respectively, while a third, the V1.1 blastomere, accounts for roughly 7% of the cells within the mature retina (Moody, 1987a). Through cell proliferation, morphogenetic movements (Li et al., 1997), and induction from the underlying mesoderm associated with gastrulation, these cells are found to reside on the surface of the embryo in an expansion known as the neural plate.

During neural plate formation, transcription factors significantly shape cellular fate decisions and migration (Saha and Grainger, 1992; Perron and Harris, 1999). Specifically, Pax6, Rx1, and Otx2 are central in this coordination (Kenyon et al., 2001). These transcription factors have been shown to modify cell identities towards a retinal fate (Mathers et al., 1997; Andreazzoli et al., 1999; Chow et al., 1999), and play a pivotal role in guiding the dispersion of cells to the eye field (Kenyon et al., 2001). They ensure that specific cells migrate to the anterior region of the embryo during gastrulation, positioning a developmentally appropriate number of them for subsequent retinal specification by other Eye Field Transcription Factors (EFTFs) at embryonic stage (st.)15 in development (Chuang & Raymond, 2001; Kenyon et al., 2001).

The formation of the eye field in *Xenopus laevis* occurs and involves coordinated cell movements leading to the specification of retinal progenitor cells (RPCs). Initially, the eye field appears as a unified crescent-shaped structure at the anterior portion of the neural plate, characterized by Pax6 and ET expression (Li et al., 1994, 1997). The mechanism by which two separate bilateral eyes form has been of historical intrigue (Stockard, 1907; Adelmann, 1929; Adelmann, 1934; Adelmann, 1936a; Adelmann, 1936b; Spemann, 1938). More recently, lineage tracing methods were used to observe that cells in the central portion of the eye field switch fates to a non-retinal lineage rather than migrate to form separate eyes (Li et al., 1997). Refining the shape of the large eye field into two separate regions has also been shown to be dependent on the eye field transcription factors Otx2, Pax6, and Rx1 (Kenyon et al., 2001). These transcription factors have been shown to convert epidermal precursors to a retinal lineage by altering their cell movements during gastrulation, allowing them to populate the native anterior neural plate (Kenyon et al., 2001). While Pax6 has been shown to be required for instantiating the developmental program for eye formation (Halder et al., 1995; Chow et al., 1999), the efficacy of Pax6 in specifying retinal cells depends on the cell’s position relative to the neural induction field, indicating the importance of the signaling environment in retinal specification (Moore et al., 2004). In addition, control of progenitor cell migration into the anterior neural plate has been shown to be instrumental in forming an appropriately sized eye. These factors include the inhibition of Fibroblast Growth Factor (FGF) and regulation by Ephrin B1 that inhibit eye-specific fates or promote the dispersal and guidance of progenitor cells to the eye field (Moore et al., 2004).

While much is known of the migration of cells into the signaling niche of the eye field less is known of the cell movements that occur within the st. 15 specified eye field. It’s generally assumed that cell movements are minimal. This assumption stems from the idea that factors promoting eye growth hold neural progenitor cells within a signaling niche, allowing the cells to take on an eye-specific fate (Zuber, 2010). These newly specified retinal progenitor cells then proliferate to form progeny which eventually fate the mature cells of the retina in a cell autonomous fashion (Rapaport et al., 2001). However, while EFTF signaling may hold progenitor cells within the eye field, the existence of such signals does not fully preclude the possibility that cells may change position within the embryonic eye before terminally differentiating into mature retinal phenotypes. However, due to the *ex vivo* nature of identifying EFTFs in fixed tissue using in situ hybridizations to locate the eye field (Zuber et al., 2003), and the limited spatial resolution of traditional cell lineage tracers (Moody, 1987a, 1987b; Eagleson & Harris, 1990), the question of whether or not cells change position in the specified eye field has not yet been answered experimentally.

In this study, we utilized a novel cell lineage tracer in a live (*in vivo*) developing embryo to investigate whether eye progenitor cells moved within the early eye field. We employed the use of a fluorescent protein, EosFP, which permanently converts from green to red when exposed to UV light, facilitating the precise labeling of small cell populations (Wiedenmann et al., 2004). A computer-controlled confocal microscopy system directed a UV laser, enabling us to create custom photoconverted regions, thereby marking retinal progenitor cells (RPCs) within the embryonic eye field in *Xenopus laevis* distinctively. This technique surpassed traditional cell tracing methods by generating a clear contrast between neighboring RPCs through selective photoconversion. We tracked the movements of RPCs within selected regions of the eye primordium during the earliest stages of eye development. This data provided here support our hypothesis that RPCs remained stationary in their region of origin within the eye field before maturing into retinal cell phenotypes.

## Materials and Methods

### Embryo Culture and Surgery

Embryos were collected following *in vitro* fertilization and cultured in 0.1X Marc’s Modified Ringer (MMR, 0.1M NaCl, 2.0mM KCl, 1mM MgSO4, 2mM CaCl2, 5mM HEPES, pH 7.8) medium (Sive et al., 2000). The embryos were incubated at 22°C until the 4-cell stage (st. 3) of embryonic development. After mRNA injection, the embryos were washed three times in 0.1X MMR, then returned to 22°C and cultured until st. 15, at which point they underwent photoconversion in 0.1X MMR chilled to 14°C to decelerate development. The photoconverted embryos were then transferred back to a 22°C incubator until reaching developmental st. 18 for imaging, and subsequently st. 35, where they were imaged and then fixed in MEMFA (100mM MOPS (pH 7.4), 2mM EGTA, 1mM MgSO4, 3.7% (v/v) formaldehyde). Post-fixation, the embryos were sectioned in the transverse plane at 60 μm using a Leica VT1000S vibratome.

### Injection of Photoconvertible Lineage Tracer

EosFP mRNA preparation, injection, and photoconversion were performed as described in Grell (in preparation), with parameters specified below. For the identification of the Nieuwkoop and Faber stage 15 (st.15) eye field, a specific point in *Xenopus* embryonic development defined by external anatomy, embryos were injected with EosFP mRNA at the concentration of 0.01 ng/nl. mRNA was generated using a template of EosFP amplified using an mMESSAGE mMACHINE kit (Ambion). Injections were directed into the dorsal D1 blastomere at the 4-cell stage, ensuring the EosFP mRNA was transcribed within cells of the neuroectoderm and the presumptive nervous system as development progressed.

The embryos were then allowed to develop to Nieuwkoop and Faber st.15 in a 14°C incubator, where the now visibly expressed green-fluorescent protein was found on one side of the embryo throughout the neural plate. st. 15 embryos were imaged and selected for their full coverage of green Eos within the anterior neural plate. This served as a large pool of embryos for photoconversions in the following sections.

### Imaging and Analysis

For imaging immediately after photoconversion at st. 15, we used a Nikon A1R confocal laser scanning microscopy system. For subsequent imaging at embryonic st. 18 and st. 35, we used a Zeiss V20 stereo fluorescent microscopy system with Zen 3.1 imaging software.

### Assessing Progenitor Cell Dynamics within the Eye Field

Our goal was to elucidate whether eye progenitor cells migrated within the eye field or differentiated directly at their point of origin. To this end, we labeled the presumptive central nervous system by injecting EosFP mRNA between .01–.003 ng/nl into dorsal blastomeres at the 4-cell stage post-fertilization. This approach ensured the neural plate expressed green Eos protein by st. 15.

We then identified, imaged, and delineated the eye fields of stage 15 embryos into four distinct spatial quadrants (anterodorsal, anteroventral, posterodorsal, posteroventral) using a confocal microscope. We photoconverted 3–5 cells within one quadrant from green to red using 408nm light, for three rounds of 5-second exposure and a 5-second dwell time between stimulations.

Following the conversion, the embryos were incubated at 14°C until they reached st. 18, at which point the eye is two cell layers thick. Embryo incubation continued to st. 35, where the mature retinal layering and differentiated cells of the retina became evident. The embryos were then fixed with a 4% formaldehyde solution for 2 hours at room temperature.

We evaluated migration or regionalization of eye progenitor cells by using fluorescent microscopy to identify the location of the red-labeled cells relative to their quadrant of origin. This procedure was repeated iteratively with different tadpoles, each with red label deposited in each of the other three eye field quadrants, to assess any differences in movement or proliferation between different regions of the eye.

### Broad Identification of Eye Field Location

**Figure 1:**
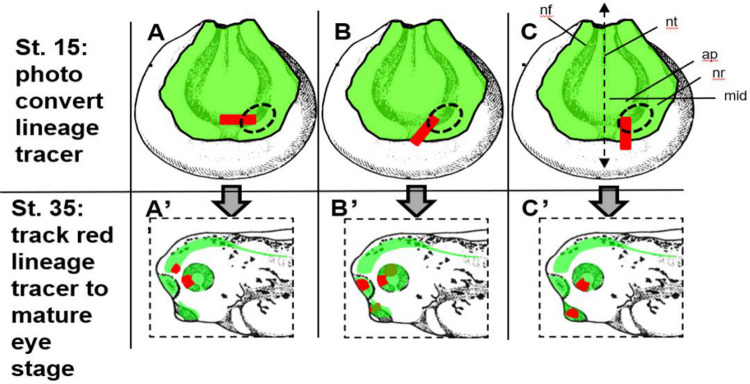
Broadly Identifying the Eye Field Border. **(A)** Broad rectangular conversion of the eye field and a region just outside of the eye field. This type of labeling was performed around the boundary of the eye field currently depicted in the literature. Broad labeling was done iteratively around the eye field **(A-C)** and tracked to st. 35 in development **(A’-C’)**. To validate the location of the eye, smaller spot conversions were made and tracked to st. 35 to confirm the exact location of the eye field in relation to physical features on the st. 15 embryo. Abbreviations: mid = midline, nt = neural tube, nf = neural fold, ap = anterior neural plate. Drawings modified from Nieuwkoop and Faber (1994).

**Figure 2:**
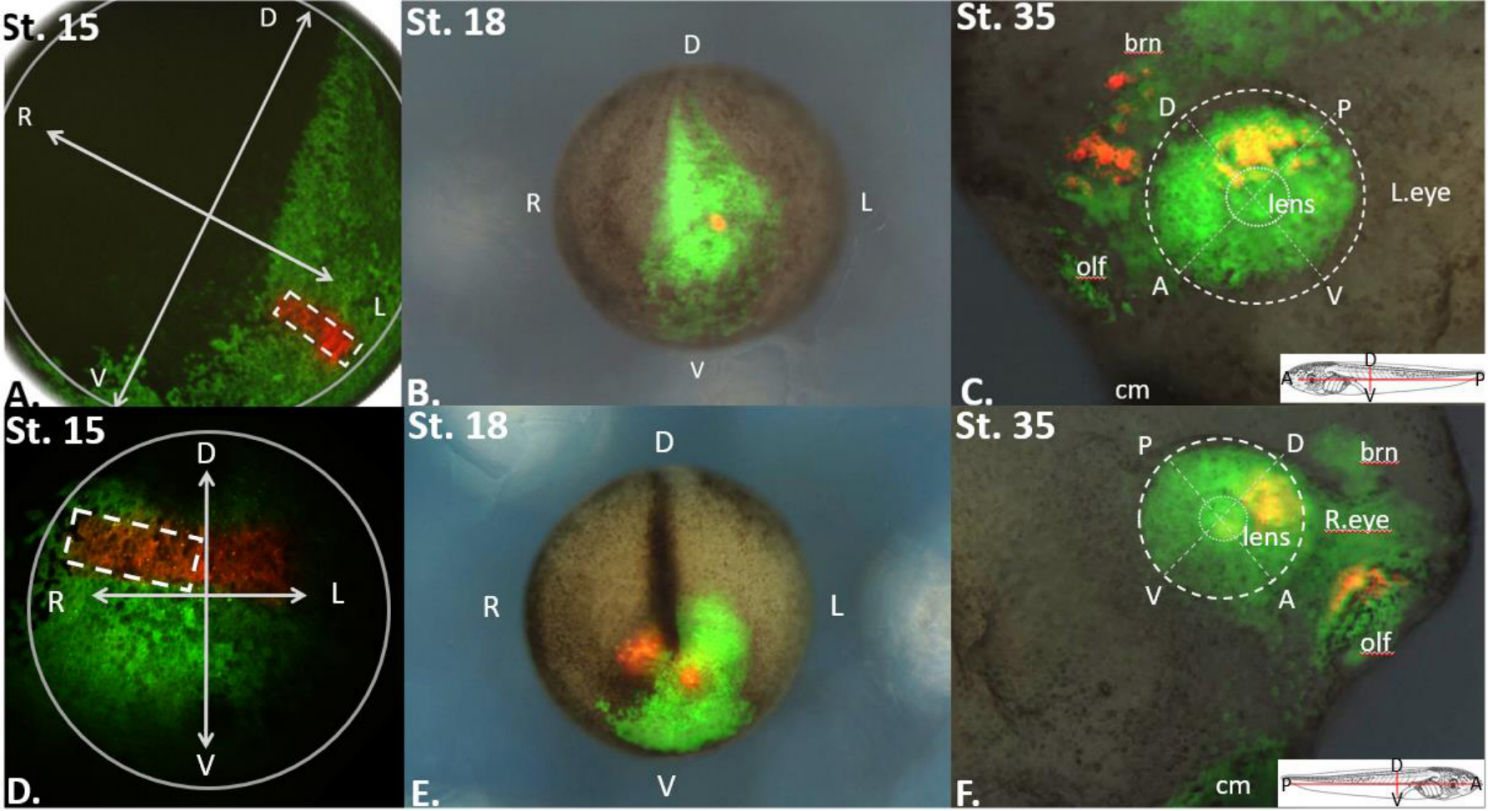
Locating the Eye Field Boundary in Relation to Surrounding Presumptive Tissue. **(A)** EosFP expressing embryos at stage 15 were photo-converted within long rectangular regions with the aim of hitting part of the eye field and a portion of the bordering tissue. **(B)** These red labeled cells were then traced to embryonic stage 18 where the label was found in the anterior portion of the presumptive eye and along the midline of the embryo. **(C)** The red label was traced until the eye took on a mature phenotype at st. 35. The red lineage tracer was found strongly in the dorsal posterior portion of the eye and the forebrain. This labeling strategy allows us to identify the eye field at stage 15 in relation to its surrounding presumptive tissues and the physical features of the embryos. **(D)** Another rectangular conversion on the anterior part of the st. 15 embryo. **(E)** The same embryo tracked to st. 18 where red labeled cells are seen partially in the right eye and midline of the embryo. **(F)** The terminal location of the red labeled cells were in the dorsal anterior portion of the st. 35 eye and the olfactory bulb. Inserts in panels C and F show orientation of the st. 35 embryo. Abbreviations: brn = brain, olf = olfactory bulb, cm = cement gland, D = dorsal, V = ventral, A = anterior, P = posterior, R= right, L= left. Drawings modified from Nieuwkoop and Faber (1994).

To delineate the eye field boundaries with our lineage tracer EosFP, we employed a two-tiered strategy involving broad rectangular photoconversions supplemented by more focused conversions. For identifying the st.15 eye field, we utilized a Nikon A1R confocal laser scanning microscopy system (UNLV confocal microscopy core). With this system, we delineated broad rectangular regions, 25 cells long by 5 cells wide, on each *Xenopus* embryo using the Nikon imaging software (Figure 1 and 2). These regions extended unilaterally into the anterior neural plate area, bound by the embryo’s midline and surrounded by the thickening neural ridge - an area previously recognized as the eye field (Zuber, 2003).

We then photo-converted our rectangular region of interest thus permanently converting the green EosFP to its red fluorescing form using a 408nm laser light. The UV laser light scanned across the selected 25×5 cell rectangular region for a 5-second exposure, followed by a 5-second dwell time. This was accomplished using a resonant scanner with a scan speed set to 100% at 30% laser power. The process was repeated for three rounds, totaling 15 seconds of light exposure and an equivalent dwell time (Figure 1 and 2).

We repeated this green to red lineage conversion for n=11 embryos at different orientations, aiming to circumscribe the boundaries of the eye field. The same set of photo-converted embryos were incubated at 14°C and allowed to develop to st. 18 and st. 35 stages, which facilitated the identification of mature structures containing the red-fluorescent cells. Upon reaching st. 35, we noted that each of the n=11 embryos exhibited two labeled structures: one identified as the eye and the other located directly outside the eye. These embryos were imaged using a Zeiss V20 upright fluorescent microscope to record the location of the labeled structures.

### Refining the Location of the st. 15 Eye Field

**Figure 3:**
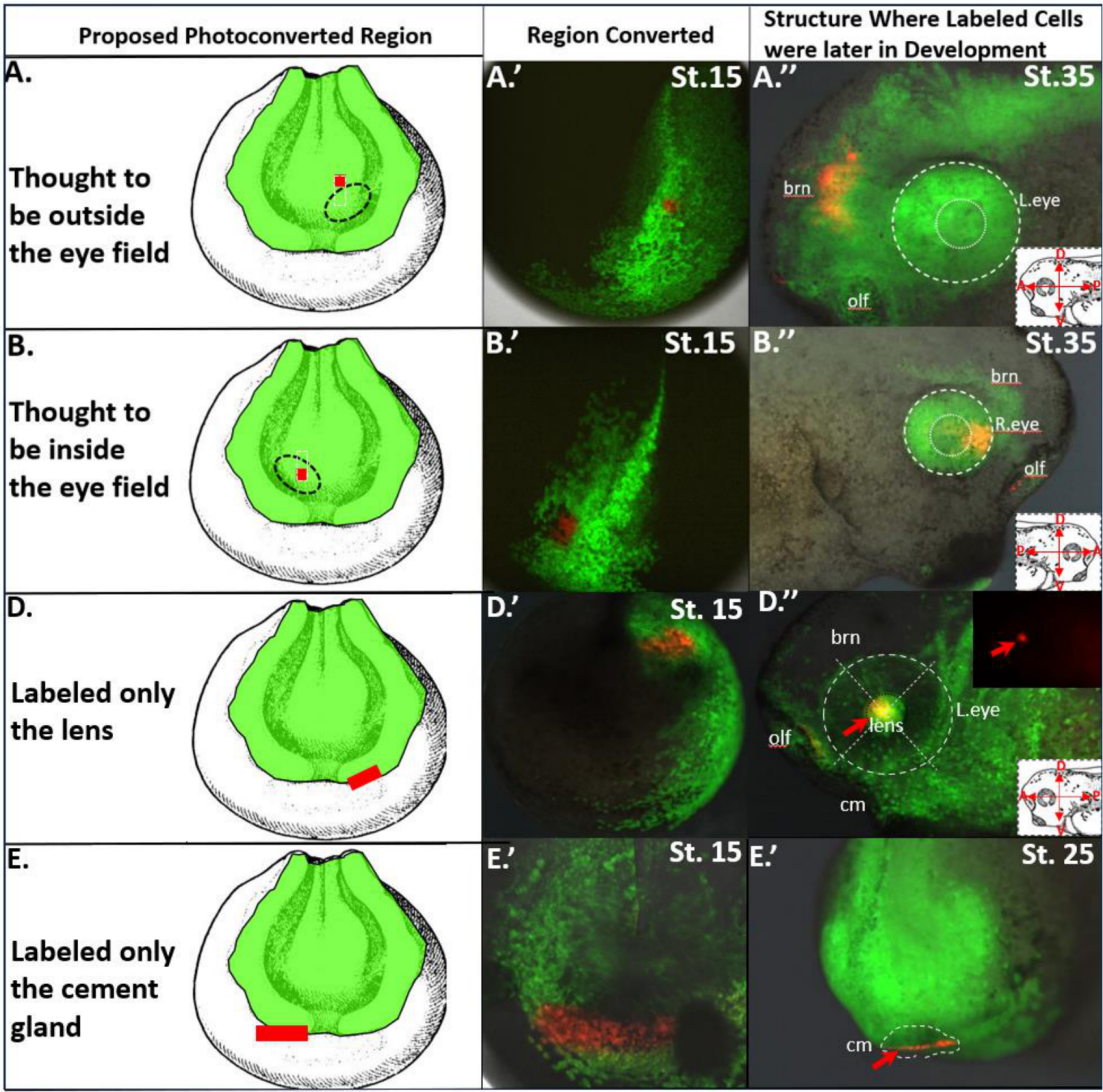
Refinement of Eye Field Location via Focal Photoconversions. **(A)** Diagram showing where a small region was photoconverted just superior where the eye fields general region was thought to be. This represents the location of a broad photoconverted region that is thought to reside outside of the eye field **(A’)** The actual stage 15 embryo that was photoconverted similar to panel A. **(A”)** The same embryo from panel A’ was allowed to mature to st. 35 where the resulting red photoconverted cells were found only within the developing brain. **(B)** Diagram showing where a small region of the anterior neural plate was photoconverted just inferior to where the photoconverted region in panel A was made but on the opposite hemisphere of another st. 15 embryo. **(B’)** The actual stage 15 embryo that was photoconverted similar to panel B. **(B”)** The same embryo from panel B’ was allowed to mature to st. 35 where the resulting red photoconverted cells were found within the eye. Using the broad photoconversions from figure 1 we were able to refine the location of the eye field, delineating where the presumptive eye was in relation to surrounding presumptive tissue regions as well as the physical features of the embryo. **(D, D’, E, E’)** Indicate the region where photoconversions were made at st. 15 in development. Although these were not focal photoconversions they only labeled one terminal tissue making them useful for forming a fate map. **(D”)** Shows where red cells were found in the lens. **(E”)** Shows the cement gland labeled from E’ at. st. 25 in development. Drawings modified from Nieuwkoop and Faber. (1994).

**Figure 4:**
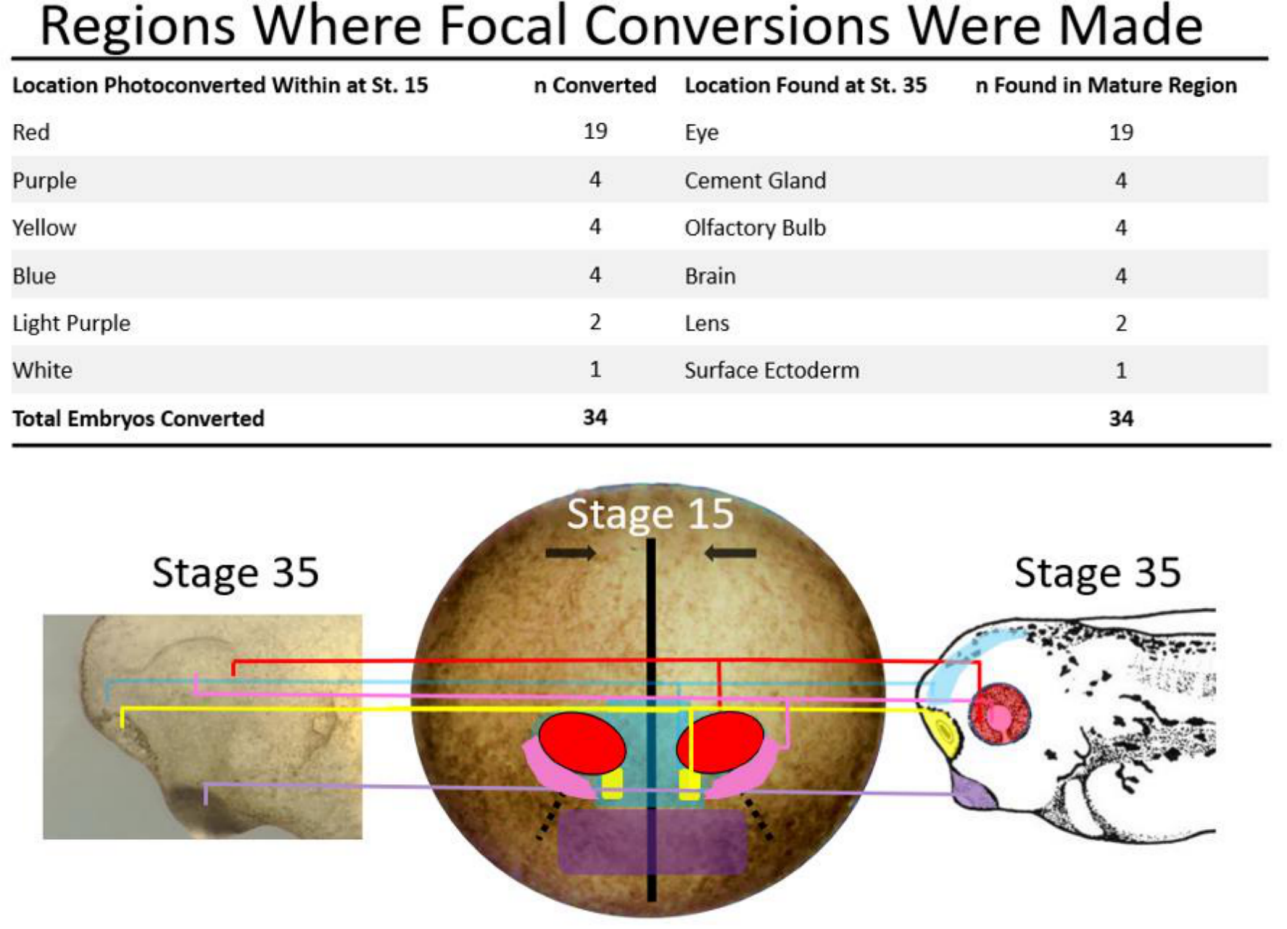
Developmental Fate Map of Stage 15 Xenopus Embryo. Each color-coded area on the stage 15 embryo represents regions where st.15 embryos were photoconverted red, and then subsequently traced to their st 35 locations. st.35 locations are seen as colors on the drawing of the st.35 tadpole and as colored lines coming from the st.15 embryo to both the drawing and brightfield image of the st. 35 tadpole. The figure describes the number of embryos photoconverted in a specific region at st. 15 and where the red lineage tracer was found at st 35. Drawings modified from Nieuwkoop and Faber (1994) Normal Table of Xenopus laevis. Drawing modified from Nieuwkoop and Faber (1994).

A precise location of the st. 15 eye field was ascertained using a series of n=34, focal photoconversions. In this approach, we conducted a comparative analysis of each side of the rectangle from our initial broad photoconversions. Each focal conversion targeted either one side or the other of a previously converted rectangle, using a separate embryo for each side (Figure 3 and 4). This included n=19 converted embryos where we thought we labeled the eye field and n=15 for the other sides of the rectangle that were thought to reside outside the eye field.

Adhering to the photo-conversion settings employed in the broader conversions, we adjusted the laser’s scannable region to a diameter of 3–4 cells for these focal photoconversions. This refinement facilitated precise tracking of the photoconverted cells to st. 18 and then to st. 35, enabling us to determine which mature tissues housed the red-labeled cells. Focused photoconversions were required to distinguish between regions that were solely within the eye or within other bordering structures.

To determine the locations of the red-labeled cells during differentiation, we imaged the embryos with a Zeiss upright fluorescence microscope. The resulting labeled structures observed at st. 35 were then recorded on a diagrammatic representation of a st. 15 embryo (Figure 4).

## Results

### Determining Eye Field Position in relation to Presumptive Tissues in the st. 15 Embryo

EosFP-expressing embryos at stage 15 were photo-converted within long rectangular regions with the aim of labeling part of the eye field and a portion of the bordering tissue (Figures 1 and 2). This is seen in Figure 2, panel A, where a red rectangle of labeled cells was photoconverted in a horizontal orientation along the anterior neural plate. These cells were then traced to embryonic stage 18, where the label was found in the anterior portion of the presumptive eye and along the midline of the embryo (Figure 2, panel B). The red-labeled cells were traced until the eye took on a mature phenotype at st. 35, as shown in Figure 2, panel C. The red lineage tracer was found predominantly in the dorsal posterior portion of the eye and the forebrain, allowing us to infer that the forebrain and the posterior dorsal portion of the eye share a common border at st. 15 in development. This labeling strategy was utilized with the rectangular photoconversion in different orientations (n=11 in total). This is seen in Figure 2, panels D-F, where the photoconverted rectangle is oriented at a 45-degree angle on the anterior neural plate in panel D. In panel E, the red cells are clustered together at the most anterior part of the folded neural tube, then within the region of the olfactory placode and anterior dorsal region of the eye at st. 35 in panel F. This suggests that cells of the olfactory placode and cells of the anterior dorsal eye share a common border at st. 15 in development. By repeating this process at 11 different orientations, we were able to determine the general location of the eye field in relation to its surrounding embryonic fields at embryonic st. 15.

Building on these broad conversions, we conducted fine focal photoconversions on two sets of EosFP-expressing embryos to pinpoint the region within the broad conversion area that was situated in the eye. This approach enabled us to locate the eye region as previously established in the literature with our lineage tracer (Moody, 1987a; Eagleson & Harris, 1990; Zuber, 2003). This process is illustrated in Figure 3, panels A and B. Panels A’ and B’ show the actual embryos where a rectangular conversion was divided into two focal converted regions in separate embryos. In panel A’, cells were converted to red in the region representing the top part of a vertical rectangle, whereas in Panel B’, cells were converted to red in the bottom region of that rectangle, albeit on the contralateral side of the embryo. The region depicted in panels B and B’ was suspected to fate cells of the eye, based on the known location of the eye field. When the red cells were traced to st. 35 in development, cells in the top portion of the vertical rectangle were found to reside within the brain, as shown in panel A”. Red-labeled cells from panel B” were discovered within the anterior dorsal portion of the st. 35 eye. Performing this procedure for each rectangle allowed us to refine the location of the eye as well as identify the presumptive tissues surrounding the eye at embryonic st. 15. This information was combined with other photoconversions that did not label the eye but were found in a single structure at st. 35. This is demonstrated in Figure 3 panels D-E”. In panel D/D’, a photoconverted rectangle was made at a 45° angle on the anterior neural ridge. These labeled cells were found only within the lens at st. 35 (Figure 3, D”). In panels E/E’, cells were labeled red in a horizontal rectangle at the anterior edge of the neural ridge. These cells were found to span the cement gland at st. 35 in development (Figure 3, panel E”). Taken together, these data indicate that the eye field resides within the anterior neural plate, surrounded by the neural ridge both rostrally and laterally.

The results from these fine focal conversions are summarized in Figure 4. Each color-coded area on the pictured embryo represents a region where individual sets of st. 15 embryos had a minimum of 3–5 cells photoconverted to red, then subsequently traced to their Stage 35 locations. The names of each colored region correspond to the tissues where the red photoconverted cells were identified at Stage 35, following the fine focal conversions within the st. 15 embryonic fields. Most notably, cells labeled in the red circular regions (n=19) consistently resided within the developing eye at stage 35. Cells labeled red in the anterior-most part of the neural ridge of the st. 15 embryo were located in the cement gland at st. 35 (n=4). Cells photoconverted in the area just posterior to the cement gland region, on the border of the neural ridge and anterior neural plate (yellow shaded regions), formed the olfactory placode at st. 35 (n=4). Cells photo-converted within the blue regions were traced to the anterior forebrain region at st. 35, while those labeled in the pink region were discovered within the lens region of the eye at later stages.

These findings provide a refined fate map of the st. 15 anterior neural plate, representing a significant contribution to future developmental research. The ability to pinpoint the exact location of the eye field, enabled by the use of our temporally persistent lineage tracer, is a powerful tool for understanding the earliest stages of eye development. This understanding is a prerequisite for conducting future regenerative studies and was instrumental in determining cell movements within the eye field in the current study.

### RPCs Within the Eye Field Differentiate in a Regionalized Manner Through Early Oculogenesis

**Figure 5:**
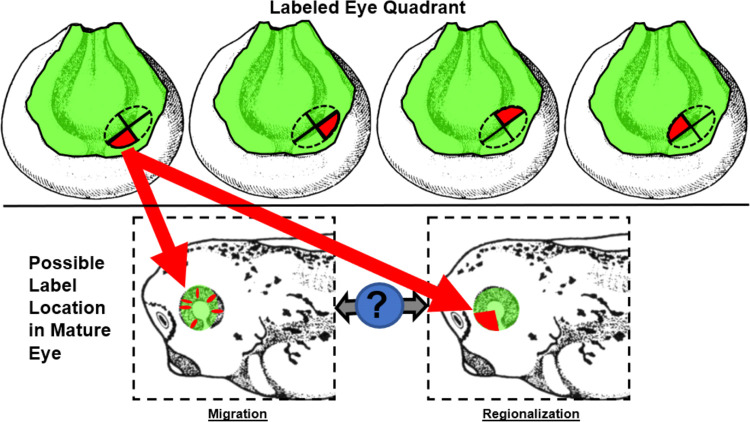
Labeling Strategy to Track RPCs During Embryonic Eye Development. Top row shows the delineation of the st. 15 eye field into 4 quadrants. Each quadrant represents a set of embryos that 3–5 cells were photoconverted within that region. The embryos from each quadrant were allowed to develop to st. 35 where the eye was more mature (bottom row) and the location of the red labeled RPCs was assessed to determine if migration or regionalized differentiation from each eye quadrant had occurred. Drawings adapted from Nieuwkoop & Faber (1994) Normal Table of Xenopus laevis.

With the st. 15 eye field location now delimited with our lineage tracer, we further sought to understand the behavior of the cells within this field. Specifically, we aimed to determine whether these cells migrate within the developing eye field or differentiate in a regionalized manner. To investigate this, we photo-converted retinal progenitor cells, located in one of the four eye field quadrants at st. 15, and then tracked these cells until they reached st. 35 (Figure 5 and 7).

**Figure 6:**
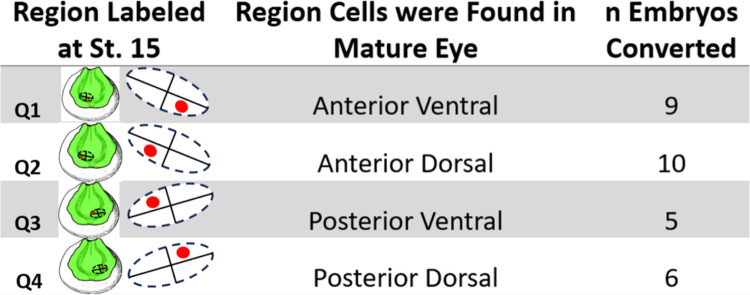
Eye Regions where Labeled Cells Were Found Clustered Together in Maturity. The drawings of st. 15 embryos indicate the region where red lineage tracer labeled 3–5 cells within a single quadrant of the eye field. The middle column denotes the location the red labeled eye cells were found at st. 35 in development. 9 embryos were converted in the quadrant depicted in the first set of drawings and found in the anterior ventral region of the eye at st. 35. Drawing modified from Nieuwkoop and Faber (1994).

**Figure 7:**
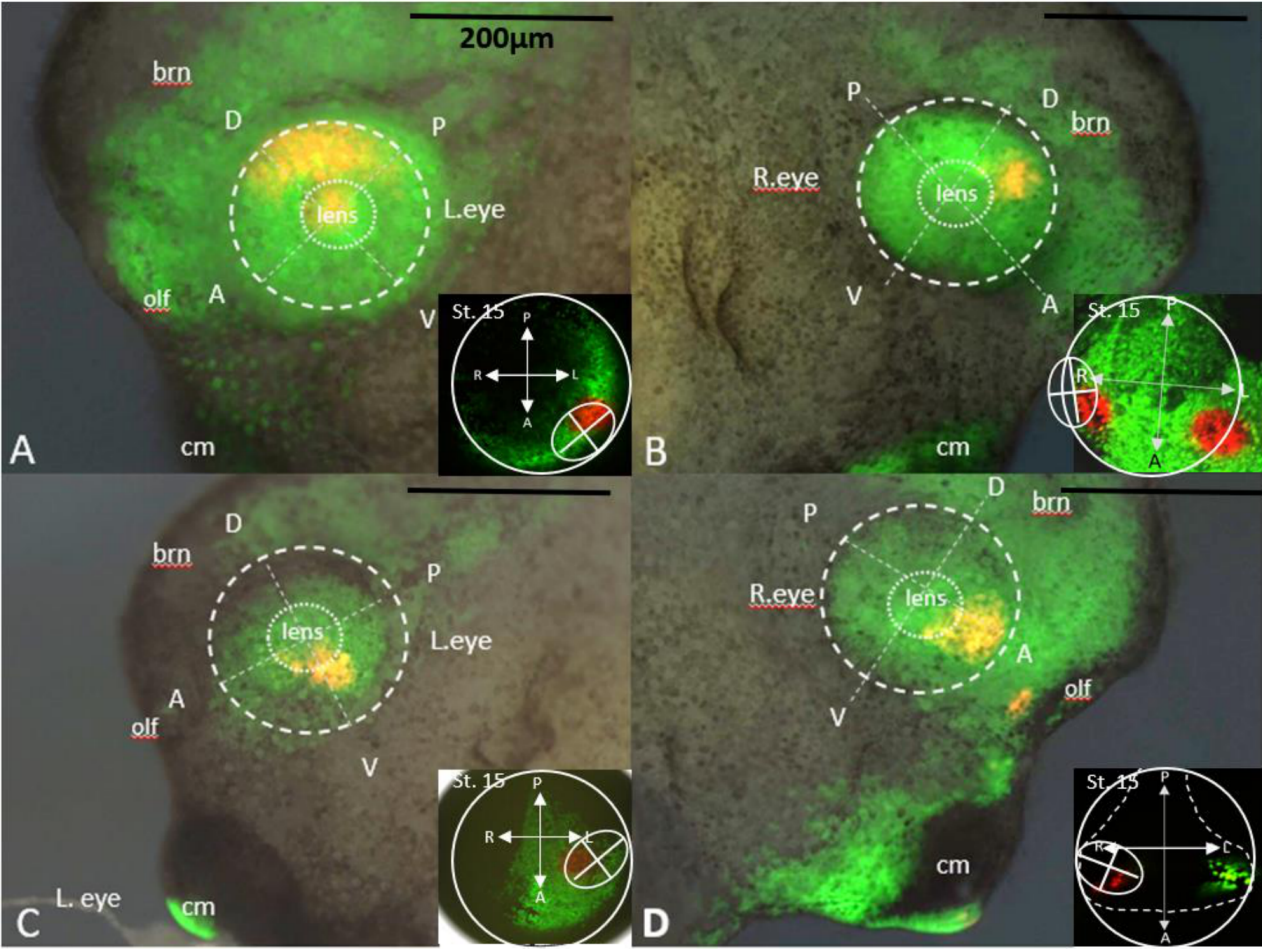
Regionalization of Eye Progenitor Cells Through Early Development. Panels **(A-D)** show the terminal location of cells labeled with one focal conversion within roughly one fourth of the eye field at st. 15 in development. Insert images for panels A, B, C, and D indicate confocal images of st. 15 embryos that were photoconverted an individual eye field quadrant. **(A)** Shows that eye progenitor cells labeled in the posterodorsal quadrant of the eye field were found clustered roughly in the posterodorsal quadrant of the st. 35 eye. **(B)** Shows that eye progenitor cells labeled in the anterodorsal related quadrant of the eye field were found clustered roughly in the anterodorsal quadrant of the st. 35 eye. **(C)** Shows eye progenitor cells labeled in the ventroposterior related quadrant of the eye field were found clustered roughly in the ventroposterior quadrant of the st. 35 eye. **(D)** Shows that eye progenitor cells labeled in the anteroventral related quadrant of the eye field were found clustered roughly in the anteroventral quadrant of the st. 35 eye. In all four regions of the eye progenitor cells were found to stay clustered together suggesting that eye progenitor cells stay regionalized during early eye development rather than migrate within the eye. Black bars represent 200 μm scale bars.

Our results are summarized in Figure 6 and 7. Q1, Q2, Q3, Q4 represent one fourth (quadrant) of the total area within the eye field. When cells were labeled red in Q1 (n=9 embryos) with a focal photoconversion, the anterior ventral quadrant of the eye contained those cells at st. 35 in development. When cells were labeled red within Q2 of the eye field, red-labeled cells were traced to the anterior dorsal region of the stage 35 eye (n=10 embryos). Similarly, when cells within Q3 were labeled red, these cells were found to reside in the posterior ventral quadrant of the eye at st. 35 (n=5 embryos). Lastly, when cells were labeled red within Q4 of the eye field at st. 15, the progeny of these cells were found to reside within the posterior dorsal quadrant of the eye at st. 35 in development (n=6 embryos).

**Figure 8:**
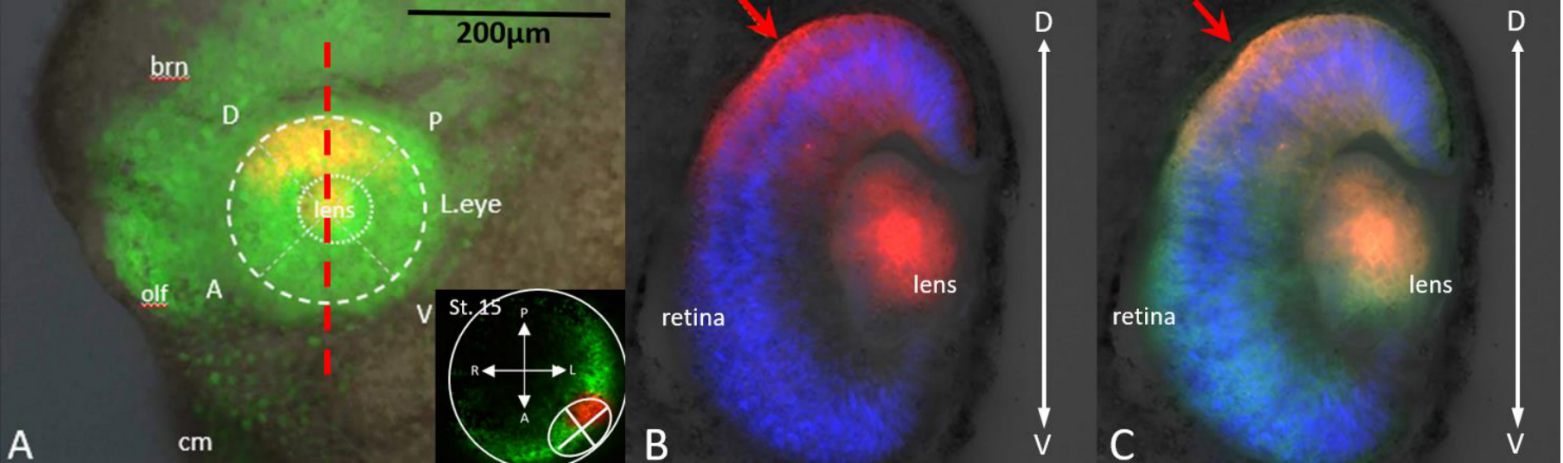
Labeled Retinal Progeny Found Throughout the Presumptive Retinal Layers. To determine if red-labeled RPCs were present throughout the depth of the developing eye, transverse sections were taken through labeled eyes at 60 μm intervals. **(A)** Shows a st. 35 tadpole containing red-labeled cells within the dorsal posterior region of the eye. The inset panel indicates the location where the RPCs were labeled at st. 15 of development. Green indicates unphotoconverted EosFP. **(B)** Depicts the distribution of red-labeled RPCs from the innermost regions of the eye, through the developing layers of the retina, to the outermost portion of the eye, indicated by a red arrow. **(C)** Presents the combined green and red channels for the same labeled left eye. DAPI-stained nuclei appear in blue, photoconverted Eos in orange, and non-photoconverted RPCs in green. Abbreviations: brn = brain, olf = olfactory bulb, cm = cement gland, D = dorsal, V = ventral, A = anterior, P = posterior. The red dashed line in **(A)** indicates the plane of transection.

Interestingly, at no point were the red labeled cells found dispersed throughout the entirety of the stage 35 eye when initially labeled from any of the four quadrants at st. 15, however, they were found clustered together in clonally related columns within the developing layers of the retina (Figure 8). These findings suggest a lack of significant cell migration within the eye field from st. 15 to st. 35, underscoring the likelihood of regional differentiation within the developing eye field. Thus, supporting our hypothesis of regionalized differentiation of RPCs within the developing eye field.

## Discussion

### Cells Maintain Embryonic Locations Within the Eye Field Post-Specification

Our research investigated the migratory behavior of Retinal Progenitor Cells (RPCs) during embryonic st. 15 eye specification to developmental st. 35 in *Xenopus* embryos. Consistent with our hypothesis, we observed that RPCs do not migrate or change location significantly within the eye field during these stages. This lack of movement is reflective of a need for a cell to be locked into a complex signaling niche for specification to a retinal cell fate, underscoring the complexities of cell fate determination mechanisms and positional signaling pathways in embryonic retinal development (Kenyon et al., 2001; Zuber et al., 2003; Moore et al., 2004). While the use of EosFP as a cell lineage tracer in eye development is robust for tracking retinal progenitor cells (RPCs), certain caveats and considerations must be acknowledged. The specificity of labeling is crucial in all lineage tracing techniques. In this study, computer-aided laser microscopy was employed to confine lineage tracer deposition to regions as small as 3–5 cells, ensuring that RPCs were labeled exclusively within the eye. However, phototoxicity is a concern with high-frequency laser use in microscopy. While we cannot exclude the possibility of unseen DNA damage in developing RPCs, embryos reared to st. 35 exhibited developmentally normal eyes and facial structures, with later stages showing normal retinal lamination, morphologically identifiable retinal cell phenotypes, and lens development (Grell, 2026), suggesting minimal developmental disruption. Another consideration is the potential dilution of the red photoconverted lineage tracer, which is not replenished and is halved with each cell division. This is particularly relevant in rapidly dividing tissues like the developing eye, leading to our decision to conclude the assay at embryonic st. 35. This timing allowed us to observe the red label’s location within the eye relative to our hypothesis, while also enabling formaldehyde fixation without quenching the Eos signal.

It is noteworthy that cell migration does occur in the differentiating vertebrate retina, typically following a radial trajectory (Morest, 1970; Nawrocki, 1985; Hinds & Hinds, 1979; Perron & Harris, 1999). In *Xenopus*, this migration pattern is seen for ganglion cells, photoreceptors, bipolar cells, and Muller glial cells (Perron & Harris, 1999), presenting as an apical-basally extending clonally related column of cells originating from the basally located RPCs. Conversely, amacrine cells, horizontal cells, and to a lesser extent some retinal ganglion cells are known to migrate tangentially from the apical-basal axis in mouse, rat, chick, and zebrafish retina (Hinds & Hinds, 1979, 1983; Prada et al., 1987; Chow et al., 2015; Icha et al., 2016). While the primary goal of this paper was to identify the migration behaviors of RPCs within the embryonic eye field, our observations extend to the point of retinal cell differentiation. From our sectioning data of the labeled st. 35 eye, we did not observe mature cell phenotypes but did note some slight dispersion from the red labeled apical-basal axis. However, this was difficult to identify since all cell progeny from red labeled RPCs were of the same color. At this point in development, the movements may have been minor but were never observed to move from the delineated eye quadrant. Future research could aim to lineage trace horizontal and amacrine cells past st. 35 to determine if tangential migration occurs at later stages in the maturing retina, assessing if this is an evolutionarily conserved process in *Xenopus laevis*.

The *Xenopus* retina, derived from a predictable subset of blastomeres (Moody, 1987a, 1987b), offers a unique model to understand the mechanisms of cellular determination and differentiation. Our study’s findings that RPCs remain within their region of inception during the eye specification period can be attributed to the influence of various signaling factors and the role of the anterior neural plate in cellular specification. Key to this process is the role of the Bone Morphogenetic Protein (BMP) pathway. The repression of BMP signaling within the animal hemisphere, as indicated by Moore and Moody (1999), is crucial for blastomeres to acquire retinal competence. This repression is instrumental in defining the domain of the presumptive neural plate, a precursor to retinal determination. This mechanism likely influences the lack of RPC migration we observed, as cells are already being positioned for their eventual fate early in development.

Additional signaling pathways, including those mediated by FGF and ephrins, are instrumental in determining the appropriate cell number and distribution within the retinogenic eye field. The formation of the eye field is driven by a series of cellular movements. The FGF and ephrin pathways are central to these processes, guiding the positioning of less lineage restricted progenitors (Wacker et al., 1998; Moore et al., 2004). Prior to gastrulation, activating the FGF pathway halts these movements within the presumptive anterior neural plate, blocking cells from assuming a retinal fate (Wacker et al. 1998). Conversely, inhibiting the FGF pathway boosts cell dispersal and adds to cell number in the eye field. Essential to this process, ephrinB1 signaling during gastrulation steers retinal progenitors into the eye field (Moore et al., 2004). Additionally, FGF signaling has the potential to alter the trajectory set by ephrinB1. The dynamic interplay between FGF and ephrin signaling ensures that the eye field within the anterior neural plate is populated with a developmentally appropriate cell count to form a normal sized eye, emphasizing the significance of these pathways in the initial stages of eye formation (Friesel and Dawid 1991; Song and Slack 1994).

Moreover, the specification of the eye field from the anterior neural plate, highlighted by transcription factors such as Pax6, rx1, and Otx2, underlines the importance of position-dependent fate determination (Hirsch & Harris, 1997; Li et al., 1997; Casarosa et al., 1997; Mathers et al., 1997). The expression patterns of these genes during gastrulation and their restriction to the eye field during later stages suggest a tightly regulated process where cells are predestined for their roles in the retina. The absence of RPC migration in our observations can be understood in this context - cells in the eye field are likely ‘locked’ into their fate early on, negating the need for migration as a means of reaching their developmental destination. Furthermore, our findings are consistent with the results from transplantation experiments of Huang and Moody (1993) and others, which demonstrate the positional dependence of blastomere progenitors in retinal specification (Huang and Moody, 1993; Gallagher et al., 1991). The contribution of blastomeres to the retina is not predetermined at the cleavage stages but is influenced by their position within the animal hemisphere. This positional influence, coupled with the signaling environment, likely restricts the RPCs to their specified locations, rendering migration unnecessary during the stages we studied.

In summary, our research underscores the complexity of retinal development in *Xenopus* embryos, particularly the interplay between signaling pathways and positional information in determining cell fate. The lack of RPC migration during eye specification and early development stages is a reflection of the intricate and highly regulated process of retinal cell fate determination.

### Identifying the Eye Field with EosFP

To delineate the eye field boundaries with our lineage tracer EosFP, we employed a two-tiered strategy involving broad rectangular photoconversions supplemented by more focused conversions. Our broad rectangular photoconversions revealed that the anterodorsal quadrant and sections of the ventral eye field at st. 15 of the anterior neural plate shared common borders with the developing forebrain along the lateral neural ridge, part of the anterior neural ridge, and medial portions of the neural plate. Furthermore, cells from the anteroventral quadrant of the eye field, the optic stalk, and the olfactory region shared common boundaries, while the cement gland was identified further rostrally on the neural ridge (Figure 4). These results are similar to previously established *Xenopus* fate maps at this point in embryonic development (Eagleson and Harris 1990, Zuber 2003). This two-tiered strategy facilitated a refined mapping of the anterior neural plate and concurrently led to a precise identification of the eye field boundaries *in vivo*. These results were vital for our project, which involved the labeling of retinal progenitor cells within the eye field, and hold implications for future research into the earliest stages of eye development. The combination of EosFP and confocal microscopy provided a reliable method to label specified eye progenitor cells early in development. The ability to track these cells *in vivo* is an advancement in our toolkit and may augment our knowledge in developmental biology, embryology, and regenerative biology research by enabling higher spatial and temporal resolution in lineage tracing experiments. Beyond *Xenopus*, our technique could be adapted to other model organisms, offering a robust tool to dissect the roles of specific cell populations in complex developmental and regenerative processes.
